# Favourable short-term course and outcome of pediatric anxiety spectrum disorders: a prospective study from India

**DOI:** 10.1186/s13034-019-0272-5

**Published:** 2019-02-28

**Authors:** Preeti Kandasamy, Satish C. Girimaji, Shekhar P. Seshadri, Shoba Srinath, John Vijay Sagar Kommu

**Affiliations:** 1 Department of Psychiatry, Jawaharlal Institute of Post Graduate Medical Education and Research, Puducherry, 605006 India; 2 Department of Child and Adolescent Psychiatry, National Institute of Mental Health and Neurosciences, Bangalore, Karnataka India

**Keywords:** Anxiety disorder, Children, Adolescent, Course, Outcome

## Abstract

**Background:**

Although anxiety disorders are the most prevalent psychiatric disorders among children and adolescents, there is a paucity of research on the course and outcome of anxiety spectrum disorders in low and middle-income countries.

**Methods:**

60 children and adolescents aged 6–16 years with anxiety spectrum disorders attending the child and adolescent psychiatry department in a tertiary care center from India were included after taking written informed consent and assent in this prospective study conducted between April 2012 to May 2014. Assessments were done at baseline, 12 weeks and 24 weeks using pediatric anxiety rating scale, clinical global impression-severity, clinical global assessment scale and pediatric quality of life scale; MINI-KID (version 6.0) was used to examine remission status.

**Results:**

Mean age of children was 12.68 years and mean duration of illness was 34.52 months. Follow-up rate at 24 weeks was 80% with a remission rate of 64.6%. Socio-demographic factors did not affect the baseline severity or course and outcome measures. Children with greater baseline severity and social phobia had a less favorable outcome at 24 weeks. Improvements made in the initial 12 weeks were maintained at 24 weeks follow up. These findings are in line with earlier studies from high-income countries.

**Limitations:**

Small sample size, attrition, rater bias.

**Conclusion:**

The study has shown a favorable outcome in children and adolescents with anxiety spectrum disorders receiving treatment-as-usual in a tertiary care setting. Adolescents who present with greater severity, comorbid with other anxiety disorders and depression at baseline require intensive intervention, and long-term follow up. There is a need for interventional research with specific focus on universal preventive programs for anxiety spectrum disorders that are feasible for delivery in low and middle-income countries.

## Introduction

Anxiety disorders are the most prevalent psychiatric disorders in children and adolescents and are considered the gateway disorders for many of the adult psychiatric disorders [1]. Anxiety disorders in adolescence predict later risks of anxiety disorder, depression, substance dependence and academic failure [2].

Epidemiological studies across the world have reported the prevalence of anxiety disorder ranging from 2 to 24% with the median prevalence rate of 8% [3–5]. Epidemiological studies from India report a prevalence ranging from 4 to 14.4% [6, 7].

The child/adolescent anxiety multimodal study (CAMS) found that response to acute-phase treatment predicted response at 6-month follow-up [8]. Children with social phobia, greater severity and comorbid depression had less favorable outcome [9–11]. Prospective studies from high-income countries report remission rate ranging from 46 to 85% [1, 12].

Studies on the course and outcome of childhood anxiety disorders are scarce in low and middle-income countries. The current study was therefore planned with the aims and objectives to prospectively study the course of anxiety spectrum disorders over 24 weeks and examine factors that modify the short-term outcome among clinic-referred children and adolescents with anxiety spectrum disorders undergoing ‘treatment as usual’ at a tertiary care center in south India.

## Methods

The study was conducted in the department of child and adolescent psychiatry at National Institute of Mental Health and Neurosciences (NIMHANS), Bangalore, which is a tertiary care center and an Institute of National importance in India offering clinical services and academic training in child and adolescent psychiatry. It has exclusive outpatient and inpatient services for children and adolescents. A multi-disciplinary team of a child psychiatrist, a clinical psychologist, and a psychiatric social worker plan and deliver evidence-based interventions. This study was conducted as part of a post-graduate dissertation and was approved by the institutional ethics committee.

Children and adolescents presenting with any subtype of anxiety disorder as per ICD 10 DCR—separation anxiety disorder of childhood (F93.0), phobic anxiety disorder of childhood (F93.1), social anxiety disorder of childhood (F93.2), generalized anxiety disorder of childhood (F93.80), social phobia (F40.1), specific phobia (F40.2), panic disorder (F41.0) were included, as were children with obsessive–compulsive disorder and post-traumatic stress disorder [13].

The study design was prospective; assessments were done at baseline and re-evaluations at 12 and 24 weeks. The study sample consisted of 60 subjects presenting to the department of child and adolescent psychiatry. Consecutive subjects fulfilling inclusion and exclusion criteria both in the inpatient and outpatient setting who consented to participate in the study during the period April 2012 to Dec 2013 were included. Subjects were included after obtaining written informed consent from parent or guardian and assent from the child.

### Inclusion criteria

1. Children and adolescents aged 6–16 years.
2. Diagnosis of any subtype of anxiety disorder, post-traumatic stress disorder and/or obsessive–compulsive disorder as per the ICD 10 Classification of Mental and Behavioral Disorders-Diagnostic Criteria for Research.

### Exclusion criteria

1. Presence of any developmental disorder as per ICD 10 DCR.
2. Presence of psychotic symptoms.

### Procedure

Children and adolescents presenting with anxiety symptoms to the child and adolescent psychiatry outpatient department were initially screened using the Screen for anxiety and related emotional disorders (SCARED) [14] followed by a detailed assessment to establish the diagnosis of anxiety disorders based on ICD 10 DCR.

There were 98 children screened during the study period and 78 fulfilled inclusion and exclusion criteria. Sixty-four parents and children gave informed consent and assent and completed the baseline assessment. Four families dropped out after the baseline assessment. Sixty children finally entered the follow-up study.

The baseline assessment included a structured interview schedule using Mini International Neuropsychiatric Interview for children and adolescents English version, 6.0 [15] and a semi-structured proforma to collect socio-demographic variables, temperament, family history, past history and treatment history. Life event scale for Indian children [16] and parent interview schedule [17] were employed to assess psychosocial adversities. Pediatric anxiety rating scale (PARS) [18] and clinical global impression [19] for severity of anxiety (CGI S); clinical global assessment scale for global functioning [20] and the pediatric quality of life scale [21] were administered at baseline and during the follow-up assessment.

The first author (PK) had prepared a workbook for cognitive behavioral interventions to standardize the interventions received by the study participants, and this was validated independently by the co-authors. The workbook included labeling anxiety, rating severity on a visual analog scale, mind–body relationship, recognizing early signs of physiological arousal, relaxation strategies, thought diary, eliciting and challenging automatic negative thought, problem-solving skills and teaching a friend overcome anxiety. The components were delivered over 8 weeks tailor made as per developmental needs of the individual child with parents serving as co-therapist.

### Statistical analysis

Descriptive statistics, repeated measures analysis of variance, one-way analysis of variance, independent samples–*t* test, Pearson’s correlation and Chi square test were employed for analysis.

## Results

The mean score on the scale SCARED child version for the 60 children who entered the study was 33.82 (SD = 12.53), and the mean score on the SCARED parent version was 28.74 (SD = 12.93), which was above the cut-off score of 25. There was a significant correlation between child and parent scores on SCARED (p < 0.01). The mean score on SCARED for the 18 children and adolescents who met inclusion criteria but refused to participate was 32.94 (SD = 10.05) and was comparable with those who participated in the study.

### Baseline characteristics

The mean age of children and adolescents in this study was 12.68 years, which was higher than the mean age of 10.7 years in the CAMS study [8]. 45% (N = 27) were female children. About 2/3 of the sample were self-referred, and 3/4 of the children were treatment-naive; the findings can therefore be generalized to a primary care setting. The mean age at onset was 9.71 years (SD = 2.24) years, and the mean duration of untreated illness was 34.52 months (SD = 3.06); lack of awareness and help-seeking attitude for internalizing disorders could explain this.

Children who were older at intake had higher severity of illness on CGI S as well as poor functioning (p < 0.05). Other socio-demographic factors, such as gender, family history or temperamental factors did not significantly affect baseline severity.

On the life event scale for Indian children, school-related stressors were elicited in 83.3% (N = 50) of children followed by interpersonal difficulties (N = 41). Though there was no gender difference in the number of life events, the stressfulness score was high for female children (p < 0.05). In nearly one-third of the sample (N = 19) school refusal was noted, 13 of these children were not schooling at the time of consultation reflecting significant impairment in academic functioning. On parent interview schedule abnormal quality of upbringing such as overindulgence, overprotection and the inappropriate parental pressure was elicited in 70% (N = 42) of the sample followed by chronic interpersonal stress related to school (N = 36). Children with more number of life events and psychosocial adversities had significantly high baseline severity on PARS and CGI S (p < 0.001).

Social phobia was the most common subtype of anxiety spectrum disorders at baseline (Table 1). Eighty percent (N = 48) of children with an anxiety disorder had a comorbid disorder. About 56.7% (N = 34) children had 2 or more anxiety disorders at baseline (Fig. 2). Comorbid depressive disorder was diagnosed in 28.3% (N = 17) of children. Presence of comorbid anxiety disorder (p < 0.05) or depressive disorder significantly increased the baseline severity (Table 2); 28.3% (N = 17) had current/lifetime occurrence of suicidality on MINI-KID.

**Table 1:** Frequency of subtypes of anxiety disorder as per ICD 10 DCR (including comorbid anxiety disorders)

ICD- 10 DCR	N	%
Social phobia	30	50
Generalized anxiety disorder	23	38.3
Obsessive–compulsive disorder	15	25
Separation anxiety disorder of childhood^a^	9	15
Phobic anxiety disorder of childhood^b^	7	11.7
Panic disorder	6	10
Social anxiety disorder of childhood^a^	5	8.3
Specific phobias	4	6.7
Post-traumatic stress disorder	3	5

^a^Onset before 6 years, an absence of generalized anxiety disorder

^b^Developmentally phase-appropriate but abnormal in degree, an absence of generalized anxiety disorder

**Table 2:** Baseline severity and comorbid depressive disorder

Variable	Comorbid depression		t	p
	Yes (N = 17)	No (N = 43)		
	Mean (SD)	Mean (SD)		
PARS^a^	24.75 (2.82)	19.86 (4.75)	− 4.867	0.000*
CGAS^b^	43.43 (11.10)	54.68 (14.02)	2.889	0.005*
CGI-S^c^	5.12 (0.88)	4.32 (1.09)	− 2.646	0.010*
PedsQL^d^	35.94 (10.04)	24.93 (12.96)	− 3072	0.003*

* p < 0.05

^a^Pediatric anxiety rating scale, ^b^Children’s global assessment scale, ^c^Clinical global impressions scale- severity, ^d^Pediatric quality of life

Comorbid attention deficit hyperactivity disorder was found in 16.7% (N = 10), oppositional defiant disorder in 13.3% (N = 8) and specific developmental disorder of scholastic skills in 23.3% (N = 14). Two children had stuttering, and 4 had other medical disorders. Age and gender did not significantly affect patterns of comorbidity.

### Course and outcome

**Fig. 1:**
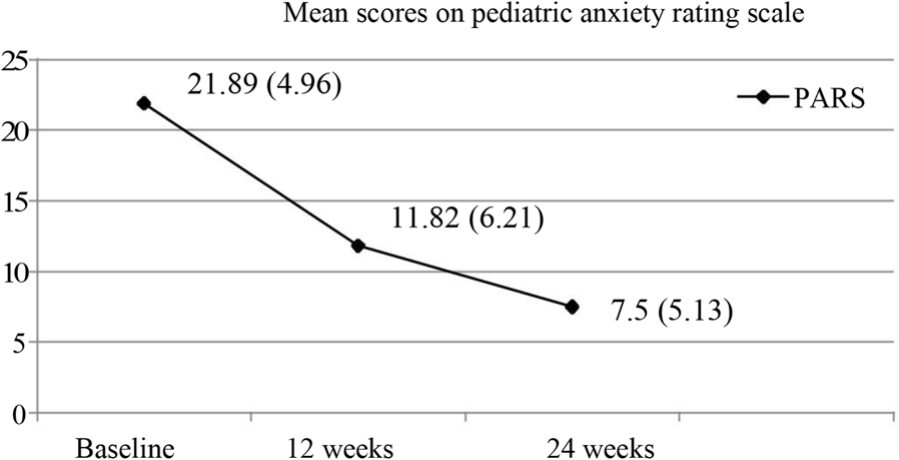
Course of anxiety disorder

**Fig. 2:**
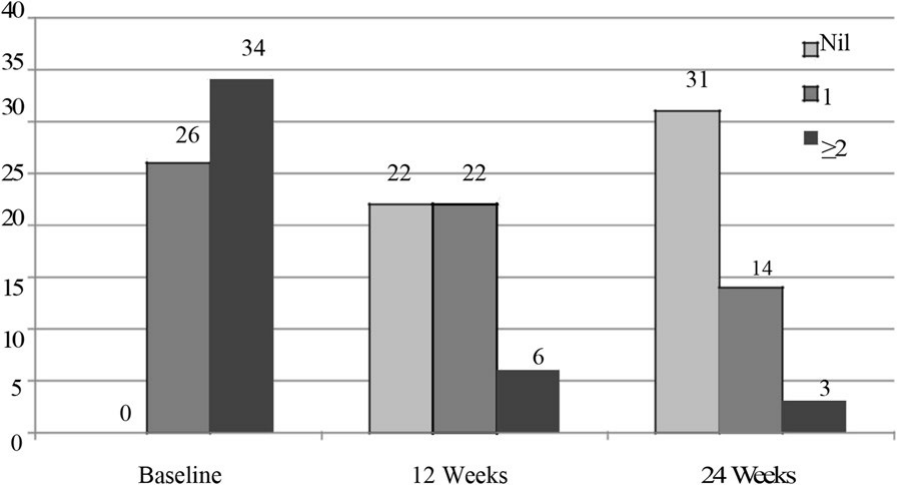
Number of children with 2 or more anxiety disorders at baseline and at follow up

Figures 1 and 2 shows the symptom severity and the number of anxiety disorder at baseline and during follow up. The mean severity scores dropped below 13 on PARS and were no more in the clinically significant range by 12 weeks [8]. There was a significant improvement in symptom severity, clinical global assessment of functioning and quality of life on repeated measures analysis of variance and a significant difference between scores was found at all three assessment points (p < 0.001).

**Fig. 3:**
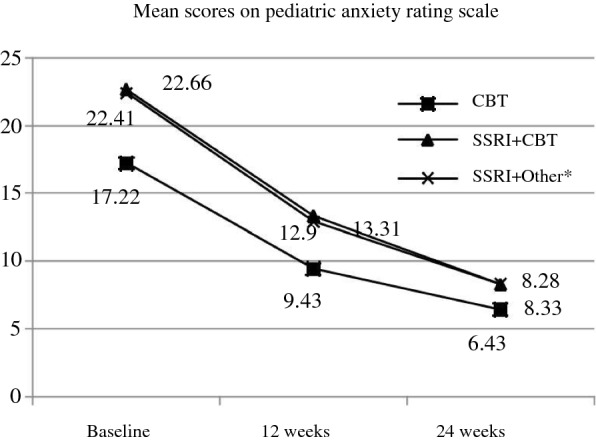
Intervention and course. *other non-pharmacological interventions

Figure 3 depicts the correlation between interventions and severity scores on pediatric anxiety scale. The modality of treatment did not affect course over 24 weeks. Baseline severity on CGI S, PARS and severity of impairment on CGAS (p < 0.001) predicted the use of pharmacological intervention.

Table 3 summarises the interventions provided to children. Among children receiving pharmacological interventions majority of them received fluoxetine; 20 children received dosage in the range 10–20 mg and those with OCD (N = 15) received higher doses of fluoxetine.

**Table 3:** Type of interventions provided

Type of intervention	N	%
Psycho-education of child and family	60	100
Cognitive behavior therapy	42	70
Pharmacological-SSRIs	40	66.7
Combined CBT and medication	32	53.3
Addressing unhealthy parenting practices	50	83.3
Working with school authorities	29	48.3
Behaviour therapy	16	26.7
Family interventions	8	13.3
Group interventions	8	13.3

Among those who completed 24 weeks follow-up, remission on MINI-KID was 64.6% and on CGI-S was 58.3%. With the assumption that all the drop-outs could have been non-responders the remission rates still range from 46.7 to 51.7% at the 24 weeks follow up.

Two children received an additional diagnosis of MDD during the 12th-week assessment, one developed dissociative disorder and another child had a hypomanic switch on SSRI. There was no attempt of self-harm or non-suicidal self-injury in the study participants during follow up.

Baseline severity and comorbidity were the only predictors of remission status at 24 weeks (Table 4). Among the subtype, social anxiety had lesser remission rates as compared to other subtypes (Table 5) and is as reported in earlier studies [11]. However, few children (N = 5) with social anxiety moved from generalized to non-generalized subtype on MINI-KID by 24 weeks reflecting a fall both in severity as well as the number of feared social situations. The workbook employed in this study used generic components; targeted interventions for social anxiety and long-term follow-up may be indicated for children with social anxiety.

**Table 4:** Predictors of remission

Baseline severity	Remission on MINI-KID			F	p
	Yes (N = 31)	No (N = 17)	NA (N = 12)		
	Mean (SD)	Mean (SD)	Mean (SD)		
PARS^a^	20.33 (4.77)	24.82 (3.63)	18.92 (4.81)	8.985	0.000**
CGAS^b^	55.23 (14.67)	44.41 (12.81)	52.88 (11.32)	3.543	0.035*
CGI S^c^	4.22 (0.96)	5.35 (0.93)	4.17 (1.11)	8.278	0.001*
PEDSQL^d^	25.03 (14.28)	32.76 (11.51)	28.25 (10.71)	1.976	0.148
Number of axis I diagnosis	2.22 (1.02)	3.35 (1.22)	2.17 (1.02)	6.794	0.002*
Number of anxiety disorders	1.68 (0.79)	2.29 (1.05)	1.58 (0.90)	3.222	0.047*

*NA* not assessed

* p < 0.05, ** p < 0.001

^a^Pediatric anxiety rating scale, ^b^Children’s global assessment scale, ^c^Clinical global impressions scale- severity, ^d^Pediatric quality of life

**Table 5:** Remission status at 24 weeks

Baseline diagnosis	N	Drop out	Not remitted at 24 weeks	Remitted at 24 weeks N (%)
Generalized anxiety disorder	23	5	2	16 (69.56)
Social anxiety disorder	35	7	10	18 (51.42)
Separation anxiety disorder	20	4	2	14 (70)
Panic disorder	6	2	0	4 (66.67)
Specific phobia	11	1	2	8 (72.72)
Obsessive compulsive disorder	15	2	4	9 (60)
Post-traumatic stress disorder	3	0	0	3 (100)

### Attrition

Fifty children and adolescents (83.3%) were available for the follow-up assessment at 12 weeks, and 48 (80%) were available for the follow-up assessment at 24 weeks. Out of those available for follow-up, 68.3% (N = 41) and 48.3% (N = 29) reported in-person at the end of 12 and 24 weeks respectively. Others were interviewed over phone; children who dropped out had a lower baseline severity on PARS compared to those who completed the 24-week follow-up (p < 0.05).

Although the 24 weeks follow-up rates are comparable to the CAMS study with 78.2% follow-up rates [8], the in-person follow-up rates were less in this study. Baseline severity was the only factor, which determined the follow-up rates (p < 0.05). Age (p = 0.694), gender (p = 0.097), modality of treatment (p = 0.941) or distance from treatment centre (p = 0.273) did not significantly affect follow-up rates.

Outcome studies in adolescent substance abuse have reported that a significant number of non-contacted subjects may overestimate the outcome results [22]. In this study, however, the outcome estimates could have been underestimated as children who dropped out had a mean baseline severity significantly less than those children who remitted by 24 weeks (p < 0.05).

## Discussion

The current study is a prospective clinic-based naturalistic follow-up study of 60 children and adolescents over 24 weeks at a tertiary care center in south India. To our knowledge, this is the first study examining the course and outcome of pediatric anxiety spectrum disorders from India. It was a single-site study with minimal exclusionary criteria. Comorbid depression or past treatment was not considered as an exclusion criterion as in other interventional studies. Multiple courses and outcome measures were used to get a comprehensive picture of symptom severity, diagnostic status, functional improvement and quality of life.

A generally favorable outcome with a sharp fall in severity score in the initial weeks following treatment was observed in the current study. Socio-demographic factors did not affect the baseline severity or course and outcome measures. Improvements made in the initial 12 weeks were maintained at 24 weeks follow up. These findings are in line with earlier studies from high-income countries [8, 11].

Socio-cultural factors could have influenced the mean duration of illness apart from differences seen in help-seeking. Children with anxiety disorders often present to primary care setting for somatic complaints. Improving awareness and sensitization regarding the need for routine screening for anxiety disorders will enable early recognition [23]. Comorbid depression and high rates of suicidality, emphasizes the need for routine evaluation of comorbidity and suicidality in children presenting with anxiety disorder [24]. However, these factors may not have affected course and outcome in view of similarity with studies done elsewhere.

Also, CBT, though developed in HIC, seems to have been well received by the children and families. Adapting interventions developed from high-income countries incorporating components relevant to the socio-cultural needs of the children in low resource setting will address the current lack of structured interventions for internalizing disorders. Drop-outs were high during the early phase; this may have important clinical implication as it demands planning of sessions in a narrow time frame to address relapse prevention effectively. Considering good outcome with treatment as usual simple non-pharmacological intervention if delivered in primary care setting would benefit the children.

Findings from this study might help in developing intervention strategies best suited for low resource settings. In this study, school-related stressors contributed to considerable stress, school refusal and academic impairment and nearly half of the children (N = 29) required liaison with school authorities as part of the intervention. Considering the high prevalence and population statistics of children in low and middle-income countries school-based treatments could address school-related stress while overcoming barriers in help-seeking [25]. Universal prevention through school-based prevention program [26] could serve as a cost-effective alternative to cater to the needs of considerable number of children in resource-limited settings. While high-income countries recognize the need for such programs, it still lacks in low and middle-income countries.

## Limitation

The rater was not blind to the diagnosis, baseline severity or the modality of treatment and this could have contributed to bias.  The sample size was rather small, included a wide age span (6–16 years) and the findings may not be reliable for specific anxiety disorders. Attrition for in-person follow-up was high, though this was offset by the availability of a sizeable proportion for telephonic interviewing. The sample was heterogeneous as it included children with obsessive–compulsive disorder and post-traumatic stress disorder, which are no longer considered under anxiety disorders in DSM-5. The study was completed in 2014 and the data is relatively old.

## Conclusion

This study has shown a favorable outcome with good response and remission rates in children and adolescents with anxiety spectrum disorders receiving treatment as usual. The mean severity scores of anxiety sharply fell to clinically insignificant levels by 12 weeks. Improvements made in the initial 12 weeks were maintained at 24 weeks follow up. These findings are in line with earlier studies from high-income countries.

Adolescents with greater severity, comorbid anxiety disorder, and depression at baseline may need intensive intervention, and long-term follow up. There is a need for interventional research focusing on non-pharmacological interventions that are feasible for delivery in low resource settings. There is also a need for school-based universal preventive programs considering the high prevalence of anxiety disorders among children.
